# News Framing and Preference-Based Reinforcement: Evidence from a Real Framing Environment During the COVID-19 Pandemic

**DOI:** 10.1177/00936502221102104

**Published:** 2022-07-07

**Authors:** Florian Arendt, Michaela Forrai, Manina Mestas

**Affiliations:** 1 University of Vienna, Austria

**Keywords:** COVID-19, news framing, selective exposure, preference-based reinforcement, reinforcing spiral

## Abstract

COVID-19 is a news issue that can be covered from many different angles. When reporting, journalists have to select, accentuate, or exclude particular aspects, which, in turn, may evoke a specific, and possibly constricted, perspective in viewers, a phenomenon termed the news-framing effect. Guided by the reinforcing spiral framework, we conducted a multi-study project that investigated the news-framing effect’s underlying mechanism by studying the dynamic of self-reinforcing effects. Grounded in a real-life framing environment observed during the pandemic and systematically assessed via a content analysis (study 1) and survey (study 2), we offer supporting evidence for a preference-based reinforcement model by utilizing a combination of the selective exposure (i.e., self-selected exposure) and causal effects (i.e., forced exposure) paradigms within one randomized controlled study (study 3). Self-selection of news content by viewers was a necessary precondition for frame-consistent (reinforcement) effects. Forced exposure did not elicit causal effects in a frame-consistent direction.

The coronavirus disease (COVID-19) pandemic is an important news issue (Dan & Brosius, 2021) that has many facets and thus can be covered from many different angles. For example, journalists can emphasize health-related consequences by repeatedly reporting the daily numbers of new infections and deaths and by highlighting the beneficial consequences of government responses, such as severe lockdown measures. However, journalists can also focus on concerns related to the detrimental, unintended side effects of these measures, such as economic repercussions or impacts on public mental health. When reporting on COVID-19, journalists must therefore select, accentuate, or exclude specific aspects of the COVID-19-related reality. In turn, this emphasis on particular aspects may evoke a specific, and possibly constricted, perspective on COVID-19 in news consumers, a phenomenon termed the *framing effect* (Entman, 1993; Reese, 2001). For example, if a TV station consistently highlights death counts (relative to the consequences of government measures on the economy) and viewers accept this frame, this may have substantial implications for viewers’ perceived threat severity, their attitudes toward government responses, or even for their own behavior.

The present paper reports on a multi-study project that investigated the framing effect during the COVID-19 pandemic. Given that studies on “real-life” news-framing effects are relatively rare, as Lecheler and de Vreese (2013) noted, the present study contributes to the literature (1) by investigating news framing in a real framing environment of utmost relevance. For example, framing effects on outcomes such as (non)compliance with government measures related to the wearing of masks or social distancing can have severe consequences for individual and public health. Thus, a thorough understanding of the role of the news media and the framing effect is essential—not only for the current pandemic, but also for future crises. More importantly, the present research also contributes to the literature (2) by providing insights regarding the news-framing effect’s *underlying mechanism*, which is relevant for theory development. Specifically, we studied the dynamic of self-reinforcing effects: Cacciatore et al. (2016) emphasized how an increasingly fragmented news environment can lead viewers toward primarily exposing themselves to content that fits with their prior views and, as a result, the framing effects in modern news environments might be limited to *preference-based reinforcement*.

Unfortunately, framing-effect studies specifically targeted at preference-based reinforcement are rare. In fact, (a) previous experimental work on framing effects (e.g., Arendt et al., 2018; Nelson et al., 1997) almost exclusively relied on *forced-exposure* designs, which tend to create a gap between theory and methodology given that forced exposure does not match with the theoretical argument that selective exposure (and thus *self-selected exposure*) is fundamental in an increasingly fragmented news environment. There are also (b) non-randomized (linkage and panel survey) studies that *observe* the interplay between news use and outcomes over time (e.g., Schemer et al., 2012; Schuck et al., 2014; van Spanje & de Vreese, 2014). Unfortunately, observational studies have their limitations regarding causal interpretations. Although these two types of design clearly contribute to our knowledge, we proffer a supplementary methodological paradigm to investigate preference-based reinforcement, aiming to combine the strengths of both. In fact, we utilized a combination of the selective exposure (i.e., self-selected exposure) and causal effects (i.e., forced exposure) paradigms within one randomized controlled study, grounded in a real-life framing environment. We provide supporting evidence for a preference-based reinforcement model and show that the *self-selection* of news content by viewers was a necessary precondition for any frame-consistent (reinforcement) effects to occur—*forced exposure* did not elicit any effects in a frame-consistent direction whatsoever.

After presenting the three empirical studies—studies 1 (content analysis) and 2 (large-scale survey) provide evidence for a framing effect-consistent correlational pattern that was the basis for the randomized controlled study (study 3)—we discuss important implications for theory development and framing-effects research practice. For example, even if a framing experiment relying on forced exposure does not find any effects at all, we can*not* conclude that there is no preference-based reinforcement occurring in real life. Whether frame exposure is operationalized as forced or self-selected can make a fundamental difference.

## News Framing

By emphasizing a subset of potentially relevant considerations related to a given topic, the news media can lead viewers to focus on these considerations when constructing perceptions, attitudes, or behavioral intentions—a phenomenon termed the *framing effect*. A conventional expectancy-value conception is a helpful framework for illustrating such news-framing effects (Chong & Druckman, 2007; Nelson et al., 1997; see also Price & Tewksbury, 1997). An outcome can be defined as the weighted sum of a series of beliefs (i.e., outcome = ∑*b_i_w_i_*), where b_*i*_ is the evaluation of belief *i* and w_*i*_ is the judgmental weight associated with that belief. A framing effect occurs when a news item increases or decreases the weight of new or existing beliefs in the formation of one’s overall judgment. This view on framing effects relies on a salience-based definition of so-called emphasis-framing effects (see Druckman, 2001)—the dominant perspective in the field (e.g., Chong & Druckman, 2007; Entman, 1993; but see Cacciatore et al., 2016).

### Preference-Based Reinforcement

Consider a study relying on a survey design—a standard approach in framing research—that found a positive correlation between the amount of exposure to differently framed news and frame-consistent beliefs. Assuming that (cross-sectional) correlations that are consistent with a framing effect are not spurious—there are two traditional interpretations of such correlative findings: a causal media effect (news exposure → beliefs) or selective exposure (beliefs → news exposure). Following this logic, if a TV station consistently emphasizes the direct health effects of COVID-19 and viewers accept this perspective, exposure may elicit a substantial causal effect on viewers’ perceptions of the severity of the threat (causal effects model). It is also possible, however, that those who perceive the severity of the threat as low will specifically select a TV station that provides news content consistent with their own prior perspective on COVID-19 (selective exposure model). Although a focus on selective exposure in the news-framing domain is relatively rare when compared to the effects perspective, previous research has already acknowledged its importance and provided supporting evidence for frame-based selection (Feldman & Sol Hart, 2018).

As a supplement to these two traditional (oversimplistic) media effect models, more complex models have been developed (e.g., Cacciatore et al., 2016; Knobloch-Westerwick, 2015; Slater, 2007). They combine both strands of previous research—selective exposure and effects. This is of utmost importance for modern-day, high-choice, fragmented news environments. In fact, Cacciatore et al. (2016) argued that rapidly changing media environments and evolving viewer behaviors within these environments have begun to enter into the current paradigm of framing-effects research. Elaborating on an argumentation provided by Bennett and Iyengar (2008), Cacciatore et al. (2016) noted that an increasingly fragmented news environment will primarily match viewers up with content that fits with their prior views and, as a result, framing effects in modern news environments might be limited to what they called *preference-based reinforcement*. Cacciatore et al. (2016) emphasized the tendency among viewers to rely on highly homophilic *self-selected* news content.

While Cacciatore et al. (2016) discussed the dynamic of self-reinforcing effects in the framing domain theoretically, the reinforcing spiral framework (Slater, 2007, 2014) offers guidance for the modeling and empirical assessment of self-reinforcing effects. Consistent with Cacciatore et al. (2016) preference-based reinforcement model of framing effects, Slater (2007) conceptualized (differently framed) news use as part of a dynamic, endogenous process combining selective exposure and causal (reinforcement) effects. Stated broadly, pre-existing perceptions, attitudes, and behaviors are conceptualized to influence news-choice decisions (and thus subsequent *self-selected* exposure to frame-consistent content), which, in turn, elicit (reinforcement) effects on perceptions, attitudes, and behaviors. As Dahlgren et al. (2019) argued, the reinforcing spiral framework is “the most relevant model” (p. 5) for empirical research on the process of selective exposure and its effects over time. Importantly for the present study, the reinforcing spiral framework suggests that “the fullest and most accurate depiction of a media effects process can typically best be modeled by *assessing both selectivity and effects within the same analysis*” (Slater, 2007, p. 282, italics added). This recommendation guided our work.

Unfortunately, framing-effect studies that simultaneously look at selectivity and effects are relatively rare. As already noted above, many previous framing studies utilized experiments in which participants watched news content while not being able to select what they would prefer to watch. This (experimental) *forced-exposure* paradigm does not adequately correspond to the previously outlined characteristics of modern news environments, as audience selectivity is not considered. According to Druckman et al. (2012), the concern related to the use of “captive participants” is “a long acknowledged but seldom addressed problem” (p. 430). Additionally, non-randomized (panel survey or linkage) studies *observe* the interplay between news use and outcomes over time. However, observational studies have well-known limitations regarding causal interpretations. We proffer a supplementary methodological paradigm to test for preference-based reinforcement that utilizes a combination of the selective exposure and causal effects paradigms. We thus examine self-selected *and* forced exposure *within* the same study.

## The Present Research

We now provide the context that stimulated the present multi-study project. Afterwards, we present our formal hypotheses and an overview of the empirical work.

### Context During the COVID-19 Pandemic

In Austria, two TV stations in particular have been accused of biased COVID-19-related news coverage by critics holding different ideological positions. Critics seemed to emphasize the role of *news commentaries*: Ferdinand Wegscheider’s *Der Wegscheider*, a weekly news program on Servus TV, has been accused of downplaying the severity of COVID-19 and exaggerating the negative side effects of severe government restrictions, thus acting as “food for covidiots” (e.g., Mark, 2020; see also Kahlweit, 2021). *Der Wegscheider* aims at providing a broader explanation of individual news events, which may especially sharpen a specific frame. In contrast to the (main evening) news program that has to (more or less) cover all the relevant (newsworthy) events of a given day, news commentaries, such as *Der Wegscheider*, can especially select, accentuate, or exclude specific new developments and decidedly comment on those that are consistent with their desired perspective. Importantly, Ferdinand Wegscheider is Director (*Intendant*) of Servus TV and is therefore also responsible for other (news) content on this station.

Of interest for the present study, news coverage on ORF has been accused of exaggerating the severity of COVID-19 and, as a “mainstream media” outlet, of being obediently “loyal to the government” and blindly supportive of severe governmental measures (e.g., Wegscheider, 2020). Similar to Wegscheider on Servus TV, Günther Mayr (Head of ORF’s science division) regularly comments on the status quo of the COVID-19 pandemic within ORF’s news coverage, on (but not limited to) the main evening news program. Mayr, who was largely unknown to the Austrian public before the pandemic, has gained great popularity due to his regular news commentaries on COVID-19. He acts as “the nation’s explainer,” as he was called on a famous radio talk show (Stöckl, 2021). If there was any pandemic-related news that needed to be explained or put into context, most of the time it was Mayr who provided a commentary on ORF.

The accusations outlined above correspond to two frames that were identified in a recent systematic review of framing research in the health domain (Dan & Raupp, 2018): Health risks covered through the *alarmist frame* are characterized by claims that exaggerate risk and potentially amplify public perceptions of risk. Conversely, the *reassurance frame* portrays the health risk as less serious, potentially implying that there is no reason for audiences to worry. The striking (accused) contrast and the (actual) heated debate led us to speculate that news commentaries in particular may be the spearhead of differently framed COVID-19 news in Austria and therefore could be an appropriate focus for the present research. Although there are, of course, numerous other journalists at both TV stations, Mayr and Wegscheider are among the most salient. Thus, we made the methodological decision to focus on these two journalists’ news commentaries, as we hypothesized that we would find substantial differences in their framing of COVID-19, ranging on a continuum from an “alarmist” to a “reassurance” framing, as outlined by Dan and Raupp (2018). Although there are important organizational and societal constraints placed on individual journalists, they can play a substantial role (see Shoemaker & Vos, 2009, for a review). The decision to focus on these two journalists is also supported by the fact that there was no omnipresent single, dominant scientist explaining COVID-19 to the Austrian public, such as Anthony Fauci in the USA or Christian Drosten in Germany.

### Formal Hypotheses and Overview of the Empirical Work

We now provide formal hypotheses and a brief overview of the empirical work. We conducted a content analysis to validate the claim that Servus TV’s and ORF’s messaging differs (study 1). Although accusations surrounding the different perspectives of these two journalists were repeatedly expressed, systematic research needs solid empirical evidence. Study 1’s content analysis thus provides the basis for the investigation of the framing effect in studies 2 (correlative pattern) and 3 (preference-based reinforcement). Thus, we did not investigate the frame-building process (see Scheufele, 1999, for a contextualization) but conducted study 1 to establish systematic evidence of differently framed news content. Based on the results of the content analysis (see below), we subsequently hypothesized a framing effect-consistent correlational pattern elicited by exposure to the news commentaries of these two TV stations on three primary outcomes: perceptions of the severity of the health threat (H1.1); political attitudes toward government responses (H1.2); and behavioral compliance with government restrictions (H1.3).

Study 2’s cross-sectional survey showed a framing effect-consistent correlation between exposure and perceived severity, political attitudes, and behavioral compliance. This pattern was the basis for the randomized controlled study that investigated the dynamic of self-reinforcing effects by utilizing a combination of the self-selection and forced-exposure paradigms (study 3). Importantly, study 2 allowed us to ground the randomized controlled study in an ecologically valid setting (i.e., a real-life framing environment). In fact, we hypothesized that a preference-based reinforcement model would explain the framing effect (H2)—tested in study 3.

## Study 1

We conducted a content analysis to systematically assess whether both TV stations relied on different frames, which was a necessary first step to provide a solid basis for studies 2 and 3. We asked whether both news commentaries actually did provide different COVID-19 framing (RQ 1). We analyzed all appearances of Günther Mayr on ORF and all episodes of *Der Wegscheider* on Servus TV over the course of 5 weeks in October and November 2020. We coded for the evaluation of threat severity, the evaluation of government response severity, and the evaluation of behavioral compliance. Due to space limitations, we provide details on the method and statistical analyses in the Supplemental Material.

### Results and Discussion

Study 1 strongly confirmed the different COVID-19 perspectives in news commentaries on Servus TV and ORF on all three outcomes, answering RQ1. The differences were substantial. However, it is important to note that it is beyond the scope of the present paper to evaluate whether ORF “exaggerated” and/or whether Servus TV “downplayed” the risks. We refrain from any normative interpretations. In fact, we use quotation marks when using the terms “alarmist” and “reassuring” to emphasize the absence of normative interpretations. What is essential for the present research is that news commentaries on both TV stations indeed provided a substantially different framing of COVID-19, ranging on the framing continuum from “alarmist” to “reassurance” (Dan & Raupp, 2018).

## Study 2

The aim of study 2 was to test for a correlation between exposure to differently framed news commentaries and frame-consistent perceptions (H1.1), attitudes (H1.2), and compliance behaviors (H1.3). Establishing a robust “real-life correlation” provides the basis for the investigation of preference-based reinforcement in study 3. We conducted a large web-based cross-sectional survey with a sample from the Austrian general population (*N* = 1,176) based on quota sampling techniques (age, gender, and education) that was bought from a commercial market research institute. Data were collected from November 17, 2020 to November 28, 2020 (i.e., immediately after the observation period of study 1’s content analysis; during the second lockdown in Austria).

### Method

#### Sample

Half of the sample was female (48.4%). Nearly half had no high school diploma (48.7%), one third had a high school diploma (31.8%), and approximately one fifth had a university degree (19.5%). Participants ranged in age between 18 and 77 years (*M* = 47.71, *SD* = 15.91). The sample roughly corresponds to the Austrian population in terms of our quota variables.

#### News exposure

We asked how often participants had watched the respective news commentaries during recent weeks. Participants were asked to choose between four possible answers: *never, only once, sometimes*, and *always or nearly always*. More individuals watched the ORF news commentary—74.1% watched it at least once (*never* = 25.9%, *only once* = 10.7%, *sometimes* = 38.7%, and *always or nearly always* = 24.7%). The Servus TV news commentary was watched at least once by 35.6% (*never* = 64.4%, *only once* = 11.2%, *sometimes* = 18.0%, and *always or nearly always* = 6.4%). This difference was expected based on ratings for these two TV stations—the public service broadcaster ORF is watched by substantially more citizens compared to the private TV station Servus TV. We dummy coded this variable for both TV stations and used the *never* option as the reference category (i.e., Dummy 1 = *only once*, Dummy 2 = *sometimes*, and Dummy 3 = *always or nearly always*), resulting in six dummies. We also used other media exposure items to disentangle the unique effects of news commentaries: We measured the number of days (0–14) people had watched the main evening news program on (1) ORF or on (2) Servus TV during the previous 2 weeks. Using the same scale, we also assessed the number of days people had read (3) a newspaper or news magazine (print or online), (4) social media posts from friends or family members, (5) social media posts from celebrities and influencers, or (6) social media posts from news media.

#### Outcomes

##### Perceived severity of the health threat

We used two items to measure the perceived severity of COVID-19 (i.e., *The coronavirus elicits severe health consequences that are often lethal; The coronavirus is very dangerous and is a serious threat*). Participants were asked to rate each claim on a 7-point scale ranging from *strongly disagree* (coded as 1) to *strongly agree* (coded as 7). We calculated the mean (*M* = 5.13, *SD* = 1.57, α = .82).

##### Favorable political attitudes toward government responses

Using the same 7-point scale, we relied on four items to measure the favorability of attitudes toward government measures related to the second hard lockdown in Austria in November 2020 (i.e., *I think that the second hard lockdown in November 2020 is absolutely justified; The second hard lockdown in November 2020 is necessary and I support it; The government’s exaggerated measures have run the economy into the ground; Too many people are struggling with job loss and financial problems due to the severe government responses*). All statements pointing to an unfavorable attitude were reverse coded (*M* = 4.14, *SD* = 1.63, α = .88).

##### Behavioral compliance

We used two items to measure compliance with severe government restrictions during the first (March and April) and the second (November) hard lockdowns. These two items were phrased to include both the first (previous) and the second (“current”) lockdown—data were collected in November 2020 (i.e., *I try to comply with government measures; Back in March and April 2020, I strictly complied with government measures; M* = 6.20, *SD* = 1.17, α = .82).

#### Statistical analysis

We relied on three hierarchical multiple regression models and predicted each of the three outcomes by age, gender, education (all in step 1), all six media exposure controls noted above (step 2), and the six dummies of ORF’s and Servus TV’s news commentary (step 3). The change in *R*^2^ in the third step assesses whether exposure to news commentary is cross-sectionally related to primary outcomes. A significant change in *R*^2^ represents a pattern that is consistent with a framing effect. The different effect size of the dummies—low (*only once*), moderate (*sometimes*), or high (*always or nearly always*) amount of exposure—approximates the study of dose-dependent effects (see Arendt, 2013). Due to space limitations, we only report effect size estimates related to news commentary (see Supplemental Tables 4–6 for the full models).

#### Ethical statement

The institutional review board of the Department of Communication, University of Vienna, approved this study (Number ID: 20201016032; dated November 3, 2020).

### Results

**Figure 1.**
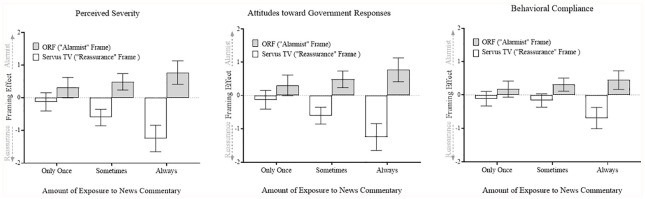
Cross-sectional association of amount of exposure to news commentary aired on two TV stations that framed the COVID-19 pandemic in different ways and three target outcomes (study 2). *Note.* Effect size estimates are based on hierarchical multiple regression models in which the amount of exposure to news commentary for both Servus TV and ORF was dummy coded (reference category = no exposure). The three dummies per TV station represent the effects of different exposure levels (i.e., *only once, sometimes*, and *always or nearly always*). All six dummies were included simultaneously within one regression model. Estimates of the framing effect (*y* axes) represent dummies’ unstandardized regression coefficients and error bars indicate their confidence intervals (95%). The full regression models can be found in Supplemental Tables 4 to 6.

We hypothesized a framing effect of exposure to these two TV stations on three primary outcomes: severity perceptions (H1.1), attitudes toward government responses (H1.2), and compliance (H1.3). Exposure to news commentary explained a significant amount of variance (step 3) in severity-of-threat perceptions, Δ*F*(6, 1,109) = 10.30, Δ*R*^2^ = .045, *p* < .001, attitudes toward government responses, Δ*F*(6, 1,109) = 12.12, Δ*R*^2^ = .052, *p* < .001, and compliance, Δ*F*(6, 1,109) = 4.77, Δ*R*^2^ = .023, *p* < .001, consistent with the hypotheses. As visualized in Figure 1, the amount of exposure mattered: Individuals who reported having watched news commentaries *only once* did not show significant effects on all three outcomes (confidence intervals overlap with zero). The strongest effects were observed for those who regularly watched news commentaries (*always or nearly always*): The more often people watched the news commentary on Servus TV (“reassurance” frame), the more they showed perceptions, attitudes, and behaviors that were more in line with a reassurance perspective (i.e., lower severity perceptions, less favorable attitudes toward government responses, and lower compliance). This claim holds for ORF, albeit in the other direction: The more often people watched news commentaries on ORF (“alarmist” frame), the more they showed perceptions, attitudes, and behaviors that were more in line with an alarmist perspective.

We also conducted an exploratory analysis: We measured exposure to the main evening news programs on ORF and Servus TV, included among all media exposure controls, as can be found in detail in the Supplemental Tables 4 to 6. Interestingly, exposure to the main evening news showed effects in the same direction as reported for news commentaries: In a regression model without news commentaries (step 2 of the hierarchical model), exposure to ORF’s main evening news increased threat perceptions, attitudes toward severe measures, and compliance, while exposure to Servus TV’s main evening news decreased all three outcomes. However, when news commentary was added in the third step of the model, the effect sizes of the main evening news substantially decreased: Servus TV’s main evening news failed to significantly predict all three outcomes. Although exposure to ORF’s main evening news program continued to elicit a significant (albeit much weaker) effect on threat perceptions and attitudes, it failed to predict compliance.

### Discussion

Study 2 provides evidence for a correlation between exposure and outcomes: Regular exposure to a specific news commentary was related to severity perceptions, attitudes toward government responses, and compliance that were more in line with the perspective presented within the respective news commentary—a correlational pattern consistent with a framing effect. This real-life correlational pattern was used as the basis for study 3. Given that different theoretical models can explain a correlational pattern like the one obtained (see our argumentation on the causal effects model, selective exposure model, and more complex models outlined above), study 3 will contribute to a more thorough understanding of this correlational pattern.

## Study 3

Study 3 tested whether a preference-based reinforcement model could explain the framing effect (H2). The dominant methodological paradigm used to study reinforcement effects over time builds on longitudinal survey designs, such as the well-known cross-lagged panel design (see Figure 1 in Slater, 2007). This approach, however, is observational and thus limited in terms of causal interpretations. Unfortunately, the best method for demonstrating causality, controlled (laboratory) experiments, has some limitations with regard to testing reinforcing spirals, as it includes forced exposure via the *random* allocation of news content instead of the *self*-selection of news content (which is needed to test for preference-based reinforcement). In the present study, we thus decided to utilize a methodological paradigm that allowed for a thorough test of the dynamic of self-reinforcing effects, including both self-selected exposure and forced exposure *within* one randomized controlled study, as Slater (2007) suggested for empirical research. Consistent with this recommendation, the design of study 3 utilized two arms: (1) a selective exposure study including subsequent (*self-selected*) exposure (aim: to test preference-based reinforcement using the self-selection of differently framed news items by viewers) and (2) a *forced-exposure* experiment (aim: to test the overall “across-the-board” causal effects by relying on the random allocation of news items). Thus, depending on the study arm, the procedure (i.e., random or participant news-item selection) was varied.

Everything was equal between both arms (*ceteris paribus*). There was only one difference between both arms: self-selection vs. forced exposure. A random assignment ensured that the sample characteristics were similar among participants in both arms. (A randomization check indicated that there were no significant differences in terms of age, gender, and education—the quota variables that were used in study 2.) This allowed for an adequate comparison between self-selection and forced exposure within one study.

### Method

#### Sample

A convenience sample from the Austrian general population was recruited via the non-commercial online access panel *SoSci* (https://www.soscipanel.de/). In total, 2,327 participants clicked on the first page of the web-based study. Of these, 1,684 participants completed the study. To increase data quality, participants who spent less than 8 minutes participating in the study were excluded based on a priori considerations (*n* = 888)—all stimulus videos were about 4-minutes long, and based on initial pre-testing, filling out the questionnaire (including reading the study introduction and providing informed consent) in under 4 minutes was deemed unlikely—resulting in a sample size of *N* = 796 for data analysis, which is consistent with *a priori* power considerations. Based on our own experience with that specific online access panel, we expected that many individuals would not watch the whole video, which is, of course, a prerequisite for any effects to occur—*SoSci* panel members do not get financial compensation. Thus, these hard exclusion criteria, defined before conducting the study, were necessary to ensure high data quality. Such exclusion criteria are also consistent with standard practices for processing and cleaning in web-based studies (Toepoel, 2016). We planned this high number of completions to ensure that we had enough participants after the exclusion process.

Approximately half of the sample was female (56.3%). The minority of the sample had no high school diploma (12.1%), about one third had a high school diploma (30.9%), and over half of the participants had a university degree (57%). Participants ranged in age between 18 and 77 years (*M* = 47.90, *SD* = 14.79). The sample roughly corresponds to study 2’s sample in terms of gender and age. However, study 3’s sample had a higher level of formal education.

#### Forced and selective exposure

As already noted, study 3 utilized two arms. After the introductory pages (including informed consent), participants were randomly allocated to one of the two arms: selective exposure (*n* = 391) and forced exposure (*n* = 405). We collected the same variables in both parts of the study; however, participants were either randomly allocated to a specific experimental group and “forced” to watch the randomly chosen news commentary (forced exposure), while in the selective exposure arm, participants were asked which of two news commentaries they preferred to watch and thus self-selected the news item.

#### Stimulus videos

We created two videos utilizing the news commentaries that were analyzed in study 1’s content analysis. We selected several parts from different episodes. The ORF video (3:50 minutes) featured ORF’s commentator Mayr and was based on content that critics of ORF’s news coverage may have perceived as “alarmist.” Conversely, the Servus TV video (4:04 minutes) featured Servus TV’s commentator Wegscheider and was based on content that may have been perceived as “reassuring” by critics of Servus TV’s news coverage. The transcripts of each video can be found in the OSMs (Supplemental Table 7). In the forced-exposure experiment, we also used a control video, as previous framing scholarship has recommended this for experimental research (Chong & Druckman, 2007). This video was similar to the intervention videos but featured content unrelated to COVID-19 (i.e., a man who spoke about the importance of drinking enough water).

We acknowledge that the Wegscheider and Mayr videos not only differ in how they frame the COVID-19 pandemic but also in other aspects (e.g., how both commentators talk, which words they use, etc.). This was a necessary side effect of our decision to rely on real material. Although this may raise internal validity concerns, study 3 was decidedly planned to emphasize external validity. It was the explicit aim to use the real commentaries that were also the focus of study 2.

#### News choice

This variable was measured only in the selective exposure arm of the study. We asked participants to choose what they would prefer to watch. First, we pointed out that there were different commentaries on COVID-19. We mentioned ORF’s Mayr and Servus TV’s Wegscheider as two examples. Second, we asked participants to think about a “typical situation during recent weeks.” We noted that we were interested in which of these two commentaries they “would have preferred to watch.” Given that study 3 was conducted in the immediate aftermath of study 2, we decided to use this retrospective focus in the formulation of study 3’s news-choice measure to allow for more targeted interpretations of the underlying mechanism of study 2’s cross-sectional findings. As TV ratings led us to expect, the majority chose ORF’s news item (83.1%).

#### Outcomes and controls

We used the same measures as in study 2 but had to adapt the compliance measure as it measured *past* behavior in study 2. As we aimed to assess whether exposure influences future behavior, we changed the items in such a way that they assessed behavioral intentions (i.e., *During the next few weeks, I will try to comply with government measures; If there is another hard lockdown in the next few weeks or months, I will strictly comply with the government measures*): severity before exposure (*M* = 5.37, *SD* = 1.54, α = .86), severity after exposure (*M* = 5.42, *SD* = 1.59, α = .90); favorable attitudes toward government measures before exposure (*M* = 4.62, *SD* = 1.53, α = .88); favorable attitudes toward government measures after exposure (*M* = 4.55, *SD* = 1.55, α = .88); compliance intentions before exposure (*M* = 5.80, *SD* = 1.43, α = .93); and compliance intentions after exposure (*M* = 5.86, *SD* = 1.40, α = .93). Descriptive statistics show similar values to those found in study 2’s sample.

We used age, gender, and education as controls. We also measured political ideology using a standard 9-point scale ranging from *left* (coded as 1) to *right* (coded as 9; *M* = 3.88, *SD* = 1.77).

#### Ethical statement

The institutional review board of the Department of Communication, University of Vienna, approved this study (Number ID: 20201207047, dated December 9, 2020).

### Results

#### Selective exposure

We used the data from study 3’s selective exposure arm to test selective exposure. We predicted dichotomous news choice (Servus TV = 0 and ORF = 1) by target outcomes measured before exposure. Using point-biserial correlations, we found moderately strong bivariate relationships between news choice and perceived severity, *r*(389) = .53, *p* < .001, attitudes toward government responses, *r*(389) = .52, *p* < .001, and compliance, *r*(389) = .48, *p* < .001. This indicates that those with higher threat perceptions, more positive attitudes, and higher compliance more frequently selected the “alarmist” ORF news item. These bivariate relationships hold when using three separate hierarchical binary logistic regression models, controlling for age, gender, education, and political orientation: perceived threat severity, *B* = 1.00, *SE* = 0.13, Wald = 61.21, *df* = 1, Odds ratio = 2.73, *p* < .001; attitudes toward government responses, *B* = 0.94, *SE* = 0.12, Wald = 61.78, *df* = 1, Odds ratio = 2.57, *p* < .001; and compliance, *B* = 0.81, *SE* = 0.12, Wald = 46.88, *df* = 1, Odds ratio = 2.25, *p* < .001. Given that this analysis did not include measures of previous exposure, one anonymous reviewer noted that the lack of inclusion may have inflated the estimation of selectivity. Therefore, we re-ran these three logistic regression models and additionally included prior regular viewing of Servus TV’s and ORF’s news commentaries, measured by the same questions as documented in study 2’s method section (i.e., six dummy variables). This analysis provided almost identical results and can be found in the OSMs (Supplemental Table 8).

Notably, when using all three target outcomes simultaneously within one hierarchical binary logistic regression model to predict news choice, severity, *B* = 0.65, *SE* = 0.16, Wald = 16.79, *df* = 1, Odds ratio = 1.92, *p* < .001, and attitudes toward government measures, *B* = 0.49, *SE* = 0.17, Wald = 8.53, *df* = 1, Odds ratio = 1.63, *p* = .003, significantly predicted news choice; conversely, compliance intentions failed to significantly predict news choice, *B* = 0.11, *SE* = 0.17, Wald = 0.45, *df* = 1, Odds ratio = 1.12, *p* = .504.

#### Preference-based reinforcement

We used data from the selective exposure arm to test for reinforcement. We relied on three separate hierarchical multiple regression models. All controls (age, gender, education, and political orientation) and the pre-measure of the respective target outcome (to control for autoregressive effects) were included in the first step. News choice (and thus *self*-selected exposure to the Servus TV or ORF commentary) was entered in the second step. In the first regression model, severity measured before exposure predicted severity measured after exposure, *B* = 0.88, *SE* = 0.02, β = .83, *t* = 36.66, *p* < .001, indicating high stability over time (autoregressive effect). Importantly, news choice (and thus *self*-selected exposure) added explanatory value, *B* = 0.60, *SE* = 0.10, β = .15, *t* = 6.32, *p* < .001. Thus, watching the news item reinforced the pre-existing perception of severity.

Similar findings were obtained for the other two variables: In the second model, attitudes toward government responses measured prior to exposure, *B* = 0.90, *SE* = 0.02, β = .87, *t* = 45.58, *p* < .001, and news choice, *B* = 0.49, *SE* = 0.08, β = .13, *t* = 6.38, *p* < .001, predicted favorable attitudes toward severe government responses measured post-exposure. In the third model, compliance measured before exposure, *B* = 0.89, *SE* = 0.02, β = .91, *t* = 47.16, *p* < .001, and news choice, *B* = 0.21, *SE* = 0.07, β = .06, *t* = 3.10, *p* = .002, predicted post-exposure compliance. Similar to the additional analysis reported above in the selective exposure section, we re-ran these three multiple regression models and additionally included prior viewing of Servus TV’s and ORF’s news commentaries. Again, the analysis (see the Supplemental Table 8) provided almost identical results and increased our confidence in the validity of the reported findings.

**Figure 2.**
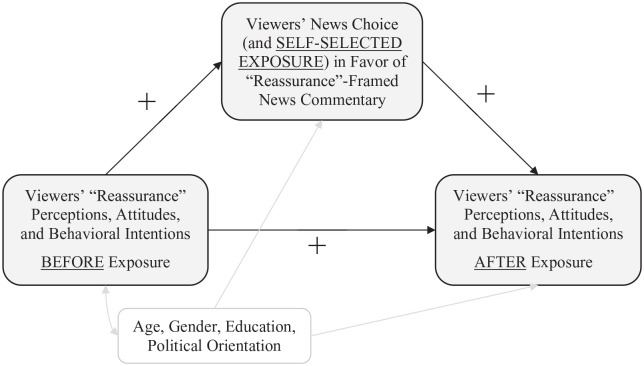
Preference-based reinforcement (study 3): visualization and effect estimates. *Note.* We used the structural equation modeling software Amos to estimate three mediator models (*df* = 0), one for each outcome. We report standardized coefficients as effect estimates: *Perceived severity*: Severity PRE → news choice: Coeff = .49, *p* < .001; news choice → severity POST: Coeff = .15, *p* < .001; direct effect: Coeff = .83, *p* < .001; indirect effect: Coeff = .07, *p* = .001. *Attitudes toward government responses*: Attitudes PRE → news choice: Coeff = .48, *p* < .001; news choice → attitudes POST: Coeff = .13, *p* < .001; direct effect: Coeff = .87, *p* < .001; indirect effect: Coeff = .06, *p* = .001. *Behavioral compliance*: Compliance PRE → news choice: Coeff = .44, *p* < .001; news choice → compliance POST: Coeff = .06, *p* < .001; direct effect: Coeff = .91, *p* < .001; indirect effect: Coeff = .03, *p* = .009.

To deepen our understanding, we performed a further analysis: We used the structural equation modeling software Amos to test preference-based reinforcement by specifying a mediator model (independent variable = outcome measured before exposure; mediator = news choice [and thus self-selected exposure]; dependent variable = outcome measured after exposure; controls: age, gender, education, and political orientation). We estimated three separate models (*df* = 0), one for each outcome. *p-*Values were based on bias-corrected bootstrapping confidence intervals (95%). A visualization and the effect estimates can be found in Figure 2. Taken together, the findings indicate that perceptions, attitudes, and behavioral intentions consistent with an “alarmist” (“reassurance”) frame influenced news choice in a frame-consistent direction, thus leading to *self-selected exposure* to a specifically framed news commentary, which in turn influenced (i.e., reinforced) perceptions, attitudes, and intentions in an even more “alarmist” (or “reassuring”) direction. This supports a preference-based reinforcement model, as predicted by H2.

#### Overall “across-the-board” causal effect

We used the data from the experimental forced-exposure arm to test for an overall “across-the-board” causal effect by asking whether forced exposure elicited causal effects in a frame-consistent direction. Note that H2 does not formally predict overall “across-the-board” causal effects elicited by forced exposure, as forced-exposure effects are not necessary in a preference-based reinforcement model (in contrast to reinforcement effects based on self-selected exposure). Unlike in study 3’s selective exposure arm, in this arm of the design, participants were *randomly* allocated to watch one news item (i.e., *forced exposure* in an experiment). We relied on three separate mixed-model analyses of variances (ANOVAs) with the outcome as the within-subjects factor (i.e., time: outcome measured before and after watching) and experimental group (news item: control, ORF, Servus TV) as the between-subjects factor. A significant causal effect of watching the video would be indicated by a significant interaction (time × group), indicating a change in the outcome in the treatment groups (relative to the control group). We did not control for third variables given that this arm of the design relied on random assignment.

There were no interaction effects for attitudes toward government responses, *F*(2, 402) = 0.52, *p* = .597, η^2^ = .003, and compliance intentions, *F*(2, 402) = 1.13, *p* = .324, η^2^ = .006. This indicates that there were no overall “across-the-board” causal effects of watching a news item on attitudes and compliance. However, analyses indicated a significant interaction effect for severity-of-threat perceptions, *F*(2, 402) = 5.05, *p* = .007, η^2^ = .024. Importantly, this interaction effect was driven by the group that watched the “reassuring” Servus TV news item. Unexpectedly, exposure to the “reassuring” Servus TV news item *increased* severity perceptions (“boomerang effect”): Severity measured after watching (*M* = 5.60, *SD* = 1.54) was higher compared to when measured before watching (*M* = 5.46, *SD* = 1.53) in those who watched the Servus TV news item, *t*(134) = –3.19, *p* = .002. There were no significant severity-related before–after mean differences in the ORF group, *t*(139) = 0.24, *p* = .814, or in the control group, *t*(129) = 1.32, *p* = .188.

### Additional Analysis: Direct Comparison of Self-Selected and Forced Exposure

Nisbet and Scheufele (2009) argued that research should develop a better understanding of how viewers “will filter or reinterpret” media content “*when it reaches them*, given their personal value systems and beliefs” (p. 1774, italics added). It is thus of relevance to understand viewers’ predispositions when starting to interpret the news content. We are thankful to one anonymous reviewer who channeled our attention to a direct comparison between those with self-selected and forced exposure. Thus, we looked at the difference in pre-measured perceptions, attitudes, and intentions between those with self-selected exposure and those with forced exposure. We merged data from the selective exposure arm and the causal effects arm of study 3’s design and used three two-factorial ANOVAs with a 2 (type of exposure: forced or self-selected) × 2 (news commentary: Servus TV or ORF) design, one for each of the three target outcomes. A difference between forced and self-selected viewers would be indicated by a significant interaction effect.

There were significant interaction effects for severity perceptions, *F*(1, 662) = 84.15, *p* < .001, η^2^ = .113, attitudes toward government responses, *F*(1, 662) = 77.09, *p* < .001, η^2^ = .104, and compliance intentions, *F*(1, 662) = 51.33, *p* < .001, η^2^ = .072. Figure OSM 1 in the OSMs provides a visualization and indicates a pattern consistent with “transverse contingent moderation” (Holbert & Park, 2019), that is, there was no difference in predispositions between news-commentary groups in those with forced exposure (due to random allocation), but there was a difference in those with self-selected exposure due to predisposition-consistent news choice. Consistent with the findings reported above, this illustrates that forced viewers and self-selectors were substantially different before watching and interpreting the news commentary. Interestingly, this was most pronounced for Servus TV. Although ORF showed a similar pattern, the difference between forced viewers and self-selectors was smaller; presumably because of a ceiling effect—study 3’s sample already showed high “alarmist” means on perceptions, attitudes, and compliance (see above).

Furthermore, we tested whether pre-measures of perceptions, attitudes, and compliance intentions moderated the effect of watching the ORF news item (vs. the Servus TV news item) on perceptions, attitudes, and compliance intentions measured after exposure *in forced viewers*. One possibility is that a predisposition-congruent frame may have strengthened outcomes regardless of whether individuals self-selected or were “forced” to watch the commentary—what may have simply counted might have been the difference in predispositions when starting to process the news content. If this idea is correct, then the experimental study (i.e., forced viewers) should indicate (conditional) causal effects in those with pre-measured perceptions, attitudes, and compliance intentions in line with the frame in the news item. We used a moderated multiple regression with the data provided by participants in the forced-exposure arm (*n* = 275) to test this idea. We predicted severity (attitudes and compliance) measured after exposure by news commentary (Servus TV = 0 and ORF = 1), prior severity (attitudes and compliance) measured before exposure, and their multiplicative interaction term. A significant interaction effect would be consistent with the idea of the presence of conditional causal effects for forced-exposure viewers. Importantly, additional analyses did not provide significant interaction effects for severity perceptions, *B* = −0.03, *SE* = 0.04, *t* = −0.95, *p* = .341, attitudes, *B* = 0.01, *SE* = 0.05, *t* = 0.23, *p* = .817, and compliance intentions, *B* = −0.01, *SE* = 0.04, *t* = −0.22, *p* = .829.

As a test for robustness, we ran additional (similar) regression models, separately testing for the effects of forced exposure to the Servus TV or the ORF news commentary (vs. the control group) in forced viewers. This is a more fine-grained analysis, as it assesses possible moderation effects for the ORF and Servus TV news items separately. For example, one of these models predicted severity measured after exposure by news-commentary exposure (control group = 0 and Servus TV = 1), severity measured before exposure, and their multiplicative interaction term. This model allowed us to assess whether the effect of watching the “reassuring” news commentary (relative to the control group) elicited conditional effects in forced viewers—similar to those obtained in self-selectors—who showed low scores on the prior severity measure (i.e., “reassuring” predispositions). We ran a total of six models (i.e., two news commentaries × three outcomes), each assessing the effect of forced exposure to one of the two news commentaries relative to the control group. Consistent with the moderation analysis presented above, none of these regression models showed a significant interaction term; Servus TV (*n* = 265): severity perceptions, *B* = −0.02, *SE* = 0.05, *t* = −0.34, *p* = .733, attitudes, *B* = −0.05, *SE* = 0.04, *t* = −1.27, *p* = .203, and compliance intentions, *B* = 0.01, *SE* = 0.04, *t* = 0.27, *p* = .790; ORF (*n* = 270): severity perceptions, *B* = −0.05, *SE* = 0.04, *t* = −1.21, *p* = .227, attitudes, *B* = −0.04, *SE* = 0.05, *t* = −0.98, *p* = .326, and compliance intentions, *B* = 0.00, *SE* = 0.04, *t* = 0.08, *p* = .940. Taken together, this additional analysis indicates that there were no conditional causal effects, even in those of the forced viewers who showed more similar (frame-consistent) predispositions (as did self-selectors). The act of self-selection seemed to be a necessary precondition for any frame-consistent effects. Frame-consistent predispositions alone were *not* sufficient to elicit frame-consistent effects.

### Discussion

Study 3 provides evidence for a model that conceptualizes exposure to differently framed news as part of a dynamic process combining selective exposure and causal (reinforcement) effects. We found that pre-existing COVID-19-related perceptions, attitudes, and behavioral intentions influenced selective exposure decisions (and thus *self-selected* exposure), which, in turn, elicited a reinforcing effect on the same target outcomes. Forced exposure did not elicit effects in a frame-consistent direction.

In fact, preference-based news choice in self-selectors (1) led to a difference in the “distribution of media exposure” (Stroud et al., 2019, p. 29; see also the concept of dilution in Arceneaux & Johnson, 2013). Indeed, there were strong differences in self-selectors and forced viewers regarding perceptions, attitudes, and compliance intentions measured before watching the news item (Figure OSM 1). In addition, (2) individuals who were “forced” to view specific news content that they likely would not have selected if they were given the chance to do so seem to have reacted differently to that news content compared to self-selectors (Stroud et al., 2019, p. 30; see also the concept of differential effects in Arceneaux & Johnson, 2013). Importantly in this regard, the Servus TV news item (“reassurance” frame) elicited a boomerang effect: Individuals showed severity perceptions that were *less* consistent with the reassurance frame after watching. At a general level, Chong and Druckman (2007) already pointed to the possibility that a specific frame could elicit an unintended consequence of causing viewers to counter-argue with the frame and form judgments that go against the perspective advocated by the frame. Similarly, van Gorp (2007) emphasized that the framing process is interactive and prone to counter-frames; viewers can (more or less) actively interpret media content. In fact, he argued that if viewers define and interpret an issue in correspondence with the perspective provided in the news content, they are likely to accept the frame. However, if viewers are in an oppositional position, as van Gorp (2007) argued, effects in a frame-inconsistent direction are more likely—which is also why frames can cause effects that journalists find hard to predict and control (Scheufele, 2000). Thus, when confronted with the news commentary of Ferdinand Wegscheider on Servus TV (who expressed a *low* perception of severity, see study 1), individuals in study 3’s sample with rather *high* severity perceptions (see study 3’s methods section) may have engaged in some form of “motivated reasoning” (Kunda, 1990) or “motivated skepticism” (Taber & Lodge, 2006). Indeed, motivated processing has already been acknowledged as an important concept in framing research (e.g., Druckman & Bolsen, 2011). Therefore, as the majority of the sample showed a different mindset than Servus TV’s Wegscheider, some form of motivated processing may have led to this boomerang effect when participants were “forced” to watch the commentary.

## General Discussion

The Austrian COVID-19 context allowed us to investigate the effects of alleged “reassurance” versus “alarmist” framing—two important health frames identified in a recent systematic review of framing research in the health domain (Dan & Raupp, 2018). We were specifically interested in the framing effect’s underlying mechanism. The content analysis of news commentaries (study 1) indicated that news commentaries from two TV stations provided a different framing of COVID-19. Study 2’s cross-sectional survey showed that those who watched the respective news commentary expressed severity perceptions, attitudes toward government responses, and behavioral compliance that were more in line with the perspective presented in the respective news commentary. This correlation is consistent with a framing effect and was observed in a real framing environment. Study 3 was based on this “real-life”-grounded pattern and investigated the underlying mechanism. Guided by the reinforcing spiral framework that recommends modeling media effects by assessing both selectivity and effects within the same study (Slater, 2007), we utilized a combination of the selective exposure (i.e., self-selected exposure) and causal effects (i.e., forced exposure) paradigms within one randomized controlled study. The findings indicated that pre-existing perceptions, attitudes, and compliance-related intentions consistent with an “alarmist” or “reassuring” perspective on COVID-19 influenced news choice. Exposure to the *self-selected* news item, in turn, elicited a reinforcing effect on target outcomes. Conversely, when participants were randomly allocated and *forced* to watch a news item, we did not observe the effects moving in a frame-consistent direction. The findings thus indicated a gradual (reinforced) shift in perceptions, attitudes, and behavioral intentions in a frame-consistent direction in self-selectors, a finding that is consistent with a preference-based reinforcement model (Cacciatore et al., 2016)—an important theoretical contribution, given that framing studies specifically targeted at preference-based reinforcement are rare.

### Self-Selected Versus Forced Exposure

Forced exposure and self-selected exposure elicited different effects: Only self-selectors showed (reinforcement) effects; forced viewers did not show frame-consistent effects at all. Although we want to emphasize that previous experimental work has shown that forced exposure can elicit substantial framing effects (see above), the present research shows that there can be a fundamental difference between the effects of self-selected versus forced exposure. On a most basic level, the evidence emphasizes that selective exposure is a fundamental process that must be considered when testing and interpreting correlational evidence of a framing effect, a claim which is consistent with evidence provided in other areas of research (Knobloch-Westerwick & Johnson, 2014). Stroud et al. (2019) emphasized that the effects of media exposure may “differ when people are given the opportunity to choose content compared to when they are forced to view it” (p. 27). Conversely, forced exposure produces “captive participants” (Druckman, 2012, p. 430) who are more likely exposed to opposing perspectives, which, in turn, may stimulate defensive processing. Indeed, the problem of forced exposure in framing experiments is “a long acknowledged but seldom addressed problem” (Druckman, 2012, p. 430). The present research offers a relatively unique and rare approach.

One important implication for the practice of framing-effects research is that even if an experiment relying on forced exposure does not find any effects, we can*not* conclude that there are no preference-based reinforcement effects occurring in the real world. This has implications for future studies, as the findings may be fundamentally different when relying on self-selection or forced exposure. Thus, it may be wise to include *both* and assess whether effects differ and, if they do, how and why. Importantly, we want to emphasize that we do *not* argue that the study of self-selected exposure is more important than the study of forced exposure or that framing experiments relying on forced exposure are useless. For example, Dahlgren (2021) recently argued that individuals using social networking sites may be exposed to content they have chosen (self-selected exposure) *and* content they have not chosen (forced exposure). Thus, both types of exposure are relevant.

The observation that there was a (reinforcement) effect in self-selectors led us to assume the (more or less) absence of defensive processing in this group. The adequacy of this explanation seems to be clearly apparent and has repeatedly been used in the literature (see above). However, we argue that this assumption cannot fully explain the different effects found for self-selectors and forced viewers in the present research: Predispositions did *not* moderate the effect of exposure in forced viewers, indicating that even those with (rather) frame-consistent predispositions, comparable to self-selectors, were *not* influenced by exposure, unlike self-selectors. The absence of conditional effects in forced viewers while self-selectors showed (reinforcement) effects is thought-provoking. We now proffer a tentative idea that may help to stimulate future work.

The difference between self-selectors and forced viewers may represent some form of *choice-supportive bias*: Although this line of research is about how individuals (mis)remember past choices and does not involve selective news-exposure decisions, there are striking similarities to the findings of the present study. When individuals select one of two options, such as two potential apartment rentals, they later tend to show a choice-supportive bias insofar as, for example, they will be more likely to attribute positive features (e.g., “sunny and bright”) to the appartement they have chosen (Mather et al., 2003; Mather & Johnson, 2000). Conversely, and this is of utmost relevance for the interpretation of the present project’s findings, when choices are randomly made for them, individuals will not show such biases (Benney & Henkel, 2006; Mather et al., 2003). Therefore, choice-supportive biases seem to occur when individuals *self-select* a given choice option but not when choices are made randomly or are “forced.” Henkel and Mather (2007) argued that the objective of a choice is to pick the best option and, after making a choice, individuals are likely to harbor the belief that the chosen option is better than the options they rejected. This literature may inform future work on the difference between self-selectors and forced viewers in framing research.

### Limitations

The present research has a number of limitations. First, we used self-report measures of past behavior in study 2 and a behavioral intention measure in study 3. Self-reports of past behavior and behavioral intentions do not necessarily equate to actual behavior. Second, the present study focused on preference-based reinforcement and thus conceptualized news choice (i.e., self-selected exposure) as a mediator variable. We did not test other mediators or moderators. For example, Chong and Druckman (2007) argued that a framing effect occurs when a news item increases or decreases the weight of new or existing beliefs. We did not investigate whether exposure changed the set of considered beliefs or their weights. Third, in contrast to study 2, study 3 relied on a convenience sample of highly educated people. This decreases the comparability of study 3’s and study 2’s findings. However, the descriptive statistics of target outcomes appeared to be similar in both studies (see above), increasing our confidence in the interpretation of study 3’s findings. Fourth, the present research focused on news commentaries. We decided to focus on this news genre since it was the target of critics who—in conjunction with our own unsystematic observations (as news consumers) before conducting this project—stated that the ORF and Servus TV news commentaries provided different frames. However, there were also framing effect-consistent patterns observed for both TV stations’ main evening news programs. Fifth, study 3 used a “one-shot” stimulus presentation of edited videos. Thus, we did not investigate the effects of cumulative exposure and the influence of competing frames (Lecheler & de Vreese, 2013) or the duration of framing effects (Baden & Lecheler, 2012). Sixth, we excluded many participants in study 3 by using hard, *a priori*-defined exclusion criteria. This was deemed necessary to ensure high data quality. Seventh, we used a dichotomous news-choice measure in study 3: Participants were not able to select a “neither of them” option and thus were not able to proceed in the study without selecting one target stimulus. Although this approach is often applied in selective exposure research (e.g., Galdi et al., 2012), it may raise external validity concerns given that individuals in real life can decide not to watch any news item at all. Eighth, study 3 relied on a standard mediator model, measuring pre-existing beliefs, attitudes, and compliance. These were used to predict news choice (mediator). The same beliefs, attitudes, and compliance were measured after self-selected exposure to the news item. However, news choice is influenced by many different factors (Knobloch-Westerwick, 2015). We only focused on the target outcomes of interest (i.e., severity perceptions, political attitudes toward government responses, and compliance). Although we controlled for age, gender, education, political orientation, and prior regular viewing of the news commentaries, other variables might also have influenced the news-choice decision. Ninth, news commentaries can be perceived as experience goods: Viewers do not know the exact content of a news commentary before watching it. One may wonder whether participants know in advance what they will get out of watching a given news commentary and whether it is congruent with their perspective on COVID-19. However, research shows, for example, that viewers have (more or less detailed) perceptions or unspecific affective reactions toward media brands (Arendt et al., 2019). However, expectations before participants’ news-choice decisions were not measured. Tenth, although we found a difference between self-selected and forced exposure in the COVID-19 context, future work should enrich our understanding by studying different topics, building confidence in the generalizability of findings. COVID-19 is a very specific research context and the mechanisms underlying framing effects may be different in other topical areas (e.g., topics for which most citizens do not have strong predispositions). Eleventh, we used only two or four items to measure the primary outcomes. Future work may use more nuanced and fine-grained multi-item measures. For example, one may argue that individuals may agree or disagree with some of the attitude items (“The government’s exaggerated measures have run the economy into the ground”) for different reasons (i.e., measures are exaggerated and/or they run the economy into the ground).

### Conclusions

Despite its limitations, the present work provides supporting evidence for framing effects in a real framing environment. We offer supporting evidence for a preference-based reinforcement model of framing effects. Of utmost relevance for future work, we found that self-selection of news content by viewers was a necessary precondition for any (reinforcement) effects to occur. More research on the difference between forced and self-selected exposure is strongly needed. We provide some possible starting points for future work. A more thorough understanding of the role of self-selection and forced exposure will hopefully enrich our theoretical understanding of the framing-effects process, including its dynamic of self-reinforcing effects.

## Supplemental Material

sj-docx-1-crx-10.1177_00936502221102104 – Supplemental material for News Framing and Preference-Based Reinforcement: Evidence from a Real Framing Environment During the COVID-19 Pandemic Click here for additional data file. Supplemental material, sj-docx-1-crx-10.1177_00936502221102104 for News Framing and Preference-Based Reinforcement: Evidence from a Real Framing Environment During the COVID-19 Pandemic by Florian Arendt, Michaela Forrai and Manina Mestas in Communication Research
