# Supplementary material for: News Framing and Preference-Based Reinforcement: Evidence from a Real Framing Environment During the COVID-19 Pandemic
Source: Communic Res. 2022 Jul 7;50(2):179–204. doi: 10.1177/00936502221102104 (PMC9922665; doi:10.1177/00936502221102104)
Supplement: sj-docx-1-crx-10.1177_00936502221102104 – Supplemental material for News Framing and Preference-Based Reinforcement: Evidence from a Real Framing Environment During the COVID-19 Pandemic [file sj-docx-1-crx-10.1177_00936502221102104.docx]

**Online Supplemental Materials Document**

**Methods of Study 1**

In the following, we provide an in-depth description of the content analysis method and results.

**Method**

***Sample***

We included all appearances of Günther Mayr on ORF and all episodes of *Der Wegscheider* on Servus TV over the course of five weeks (October 24, 2020, to November 21, 2020), leading to a total of 17 appearances by Mayr and five full episodes of *Der Wegscheider*. The material from ORF was available in the form of transcripts, while the material from Servus TV was available as videos. Since the respective material was available in different formats, we had to define the cases separately. The question regarding the appropriate unit of analysis is often difficult to answer, especially for researchers who attempt to analyze both written and audiovisual content (Graber, 1989) and recent methodological research on the content analysis of frames emphasizes the question of whether to code news items all at once (e.g., a full-length interview) or as truncated sub-units, such as single answers noted within one paragraph of interview transcripts, with a preference for the latter due to higher precision (Dan, 2018). Mayr’s commentaries on ORF were based on interviews—each interview included a different number of answers—, therefore *each answer* he gave was defined as one unit of analysis, leading to a total of 34 cases for Mayr (within 17 interviews). As Dan (2018) outlined in detail, the methodological approach of truncation accurately reflects the fact that dominant frames can change *within* one news item immediately. Defining the full-length interview as the unit of analysis may be problematic, as, following Dan’s (2018) argumentation, this strategy is unlikely to provide an accurate representation of news on contested topics (Choi & Lee, 2006), such as COVID-19, in which the frames can immediately change between sub-units (e.g., between subsequent answers within an interview).

Wegscheider’s commentaries on Servus TV were monologues within episodes, available as video material. Each episode had a length of several minutes. Importantly, we could not use questions as reference points. Thus, we needed another approach to ensure that the Mayr and Wegscheider samples were comparable. As a methodological decision, each episode was split into 1-minute segments, broadly aiming to match the duration of Mayr’s given case definition. (Initial pre-testing revealed that reading one typical Mayr answer aloud took approximately 1 minute.) Although we acknowledge that this is an imperfect proxy to match the Mayr case definitions, this case-identification strategy resulted in 37 cases for Wegscheider’s commentaries on Servus TV—a comparable number. Importantly, all cases were coded independently; however, the context of the previous segment(s) was taken into account if there were references to points that were made previously. We coded a total of *N =* 71 cases (*n* =34 cases for ORF, *n* = 37 cases for Servus TV*).*

***Variables***

**Evaluation of threat severity.** We coded the commentator’s evaluation of the severity of the threat (i.e., direct health effects). Both explicit (e.g., overt, manifest) and implicit (e.g., irony) evaluations were used for coding decisions (see Früh, 2007). We assessed whether the threat severity was not mentioned, mentioned but not evaluated, evaluated as low (e.g., equating the symptoms of COVID-19 with those of the flu), or evaluated as high (e.g., mentioning that hospitals may approach their maximum capacities). If the threat severity was evaluated as both high and low within one case, we coded no evaluation, unless one was clearly emphasized.

**Evaluation of government response severity.** Similarly, we analyzed the evaluation of severe government responses (e.g., issuing a lockdown, closing down all non-essential businesses) starting in March, which marked the start of severe government measures in Austria, up until the broadcast of the news commentary. We coded government responses as not mentioned, mentioned but not evaluated, evaluated negatively (e.g., indicating that the economic consequences of government responses were too high), or evaluated positively (e.g., indicating that severe measures led to decreasing case numbers).

**Evaluation of behavioral compliance.** We also focused on the evaluation of compliance with government responses. We differentiated between no mention of compliance, mentioned but not evaluated, evaluated as unimportant (e.g., questioning the wearing of masks), and evaluated as important (e.g., emphasizing that measures can only be effective if a large number of citizens adhere to them).

***Reliability Test***

All material was coded by two independent coders. As indicated by Krippendorff’s alpha, coding was reliable for severity (α = .85), government responses (α = .84), and compliance (α = .77). After the reliability test, a final datafile was created for analysis. Disagreements in codings were solved via interpersonal discussion (consensus).

***Statistical Analysis***

We relied on a cross-tab analysis: 2 (ORF or Servus TV) × 4 (outcome: not mentioned, mentioned but not evaluated, evaluated in a “reassuring” direction, evaluated in an “alarmist” direction). We report χ² tests and Cramer’s *V* as measures of effect size. In addition, we ran Fisher’s exact tests given that the sample size was small and numerous cells had fewer than five observations. Furthermore, column proportion tests (i.e., *z* tests) using the Bonferroni correction were conducted (i.e., ORF vs. Servus TV). Due to space limitations, detailed analyses are reported in the OSMs (Tables 1–3).

**Results**

The threat severity evaluations differed significantly between the TV stations, χ² (3, *N =* 71) = 47.18, Cramer’s *V* = .82, *p* < .001. ORF’s journalist mainly (85.3%) evaluated the threat severity as high and never as low. Conversely, Servus TV’s commentator did not mention the threat in most cases (59.5%), and if he did, he mostly evaluated it as low (32.4%).

Similarly, there was a substantial difference regarding the evaluation of government responses, χ² (3, *N =* 71) = 32.32, Cramer’s *V* = .68, *p* < .001. ORF’s journalist evaluated severe government responses positively (50%) or did not mention them (32.4%). Conversely, Servus TV’s journalist evaluated severe government responses negatively most of the time (62.2%) and never evaluated them positively.

Finally, there was a significant difference regarding the evaluation of compliance, χ² (2, *N =* 71) = 35.05, Cramer’s *V* = .70, *p* < .001. ORF’s journalist either evaluated compliance as important (50%) or did not mention it at all (50%). Conversely, Servus TV’s journalist either evaluated compliance as unimportant (50%) or did not mention it at all (50%). All Bonferroni-corrected pairwise comparisons on “alarmist” and “reassuring” evaluations differed significantly between Servus TV and ORF for all three outcomes (see OSMs, Tables 1–3).

**Table OSM 1**

| *Chi-square Statistic of Evaluation of* Severity of the Threat *Among News Commentaries by Günther Mayr on ORF or by Ferdinand Wegscheider on Servus TV (Study 1)* | | | | | | | | | | | |  |
| --- | --- | --- | --- | --- | --- | --- | --- | --- | --- | --- | --- | --- |
|  | | *n* |  | Evaluation of Severity of Threat | | | |  | χ²  (df) | *Cramer’s*  *V* | *p* | |
|  |  |  |  | Threat not Mentioned | No Evaluation of Severity | Severity of Threat Evaluated as Low | Severity of Threat Evaluated as High |  |  |  |  |  |
| News Commentary | |  |  |  |  |  |  |  | 47.18  (3) | .82 | <.001 | |
|  | Günther Mayr on ORF | 34 |  | 5_a_ | 0_a_ | 0_a_ | 29_a_ |  |  |  |  | |
|  | Ferdinand Wegscheider on Servus TV | 37 |  | 22_b_ | 1_a_ | 12_b_ | 2_b_ |  |  |  |  | |
| Totals | | 71 |  | 27 | 1 | 12 | 31 |  |  |  |  | |
| *Note.* Subscript letters indicate pairs in a column (Mayr vs. Wegscheider) that are significantly different (*p* < .05; using Bonferroni correction). Reading example: There is a significant difference between Mayr on ORF (29) and Wegscheider on Servus TV (2) regarding a high evaluated severity of threat. Thus, Mayr evaluated the threat to be high significantly more often when compared to Wegscheider.  The table reports a χ² test. Given that numerous cells had fewer than five observations, we also calculated a Fisher's exact test, which supports the conclusions reported above: *p* < .001. | | | | | | | | | | | |  |

**Table OSM 2**

| *Chi-square Statistic of Evaluation of* Government Responses *Among News Commentaries by Günther Mayr on ORF or Ferdinand Wegscheider on Servus TV (Study 1)* | | | | | | | | | | | |
| --- | --- | --- | --- | --- | --- | --- | --- | --- | --- | --- | --- |
|  | | *n* |  | Evaluation of Government Responses | | | |  | χ²  (df) | Cramer’s *V* | *p* |
|  |  |  |  | Government Responses not Mentioned | No Evaluation of Severity | Positive Evaluation of Government Responses | Negative Evaluation of Government Responses |  |  |  |  |
| News Commentary | |  |  |  |  |  |  |  | 32.32  (3) | .68 | <.001 |
|  | Günther Mayr on ORF | 34 |  | 11_a_ | 3_a_ | 17_a_ | 3_a_ |  |  |  |  |
|  | Ferdinand Wegscheider on Servus TV | 37 |  | 11_a_ | 3_a_ | 0_b_ | 23_b_ |  |  |  |  |
| Totals | | 71 |  | 22 | 6 | 17 | 26 |  |  |  |  |
| *Note.* Subscript letters indicate pairs (Mayr vs. Wegscheider) that are significantly different (*p* < .05; using Bonferroni correction). Reading example: There is a significant difference between Mayr on ORF (17) and Wegscheider on Servus TV (0) regarding a positive evaluation of government responses. Thus, Mayr evaluated government responses in a positive way significantly more often when compared to Wegscheider.  The table reports a χ² test. Given that numerous cells had fewer than five observations, we also calculated a Fisher's exact test, which supports the conclusions reported above: *p* < .001. | | | | | | | | | | | |

**Table OSM 3**

| *Chi-square Statistic of Evaluation of* Compliance *Among News Commentaries by Günther Mayr on ORF or Ferdinand Wegscheider on Servus TV (Study 1)* | | | | | | | | | | | |
| --- | --- | --- | --- | --- | --- | --- | --- | --- | --- | --- | --- |
|  | | *n* |  | Evaluation of Compliance | | | |  | χ²  (df) | *Cramer’s*  *V* | *p* |
|  |  |  |  | Compliance not Mentioned | No Evaluation of Compliance | Evaluation of Compliance as Important | Evaluation of Compliance as Unimportant |  |  |  |  |
| News Commentary | |  |  |  |  |  |  |  | 35.05  (2) | .70 | <.001 |
|  | Günther Mayr on ORF | 34 |  | 17_a_ | 0_a_ | 17_a_ | 0_a_ |  |  |  |  |
|  | Ferdinand Wegscheider on Servus TV | 37 |  | 19_a_ | 0_a_ | 0_b_ | 18_b_ |  |  |  |  |
| Totals | | 71 |  | 36 | 0 | 17 | 18 |  |  |  |  |
| *Note.* Subscript letters indicate pairs (Mayr vs. Wegscheider) that are significantly different (*p* < .05; using Bonferroni correction). Reading example: There was no significant difference between Mayr on ORF (17) and Wegscheider on Servus TV (19) regarding cases in which compliance was not mentioned. The table reports a χ² test. Given that numerous cells had fewer than five observations, we also calculated a Fisher's exact test, which supports the conclusions reported above: *p* < .001. This cross-tab analysis uses *df* = 2 given that “no evaluation of behavioral compliance” was ever coded. | | | | | | | | | | | |

**Table OSM 4**

| *Hierarchical Multiple Regression Analyses Predicting Perceived Severity of the Threat from “Alarmist” (ORF) and “Reassurance” (Servus TV) News-Commentary Exposure (Study 2)* | | | | | |
| --- | --- | --- | --- | --- | --- |
| *B SE* β *p* | | | | | |
| *Step 1: Demographics (*∆*R*² = .03, *p* < .001)  Age  Gender  Education Dummy (high school)  Education Dummy (university degree)  *Step 2: Media Variables (*∆*R*² = .11, *p* < .001)  Age  Gender  Education Dummy (high school)  Education Dummy (university degree)  Main Evening News (ORF)  Main Evening News (Servus TV)  Newspapers (Print or Online)  Social Media Posts (Friends & Family)  Social Media Posts (Celebrities)  Social Media Posts (News Media)  *Step 3: News Commentary* (∆*R*² = .05, *p* < .001)  Age  Gender  Education Dummy (high school)  Education Dummy (university degree)  Main Evening News (ORF)  Main Evening News (Servus TV)  Newspapers (Print or Online)  Social Media Posts (Friends & Family)  Social Media Posts (Celebrities)  Social Media Posts (News Media)  ORF News-Commentary Dummy (only once)  ORF News-Commentary Dummy (sometimes)  ORF News-Commentary Dummy (always)  Servus TV News-Commentary Dummy (only once)  Servus TV News-Commentary Dummy (sometimes)  Servus TV News-Commentary Dummy (always) |  | 0.02  –0.24  0.10  0.10  <0.01  –0.27  –0.01  –0.01  0.09  –0.04  0.04  –0.03  0.01  0.01  0.01  –0.21  <0.01  –0.05  0.04  0.02  0.04  –0.02  0.01  0.01  0.31  0.49  0.77  –0.13  –0.61  –1.25 | <0.01  0.09  0.11  0.13  <0.01  0.09  0.10  0.12  0.01  0.01  0.01  0.01  0.01  0.01  <0.01  0.09  0.10  0.12  0.01  0.02  0.01  0.01  0.01  0.01  0.16  0.13  0.18  0.14  0.13  0.21 | .16  –.08  .03  .03  .04  –.08  <.01  <.01  .30  –.09  .15  –.09  .02  .04  <.05  –.07  <.01  –.01  .14  .04  .14  –.08  .02  .04  .06  .15  .21  –.03  –.15  –.20 | <.001  .010  .364  .418  .194  .003  .961  .944  <.001  .002  <.001  .011  .669  .345  .118  .018  .965  .701  .002  .228  <.001  .016  .536  .298  .055  <.001  <.001  .372  <.001  <.001 |
| *Note.* Gender: 1 = female, 2 = male | | | | | |

**Table OSM 5**

| *Hierarchical Multiple Regression Analyses Predicting Attitudes Toward Government Responses from “Alarmist” (ORF) and “Reassurance” (Servus TV) News-Commentary Exposure (Study 2): Results of the Multiple Regression Analysis (Study 2)* | | | | | |
| --- | --- | --- | --- | --- | --- |
| *B SE* β *p* | | | | | |
| *Step 1: Demographics (*∆*R*² = .02, *p* < .001)  Age  Gender  Education Dummy (high school)  Education Dummy (university degree)  *Step 2: Media Variables (*∆*R*² = .13, *p* < .001)  Age  Gender  Education Dummy (high school)  Education Dummy (university degree)  Main Evening News (ORF)  Main Evening News (Servus TV)  Newspapers (Print or Online)  Social Media Posts (Friends & Family)  Social Media Posts (Celebrities)  Social Media Posts (News Media)  *Step 3: News Commentary* (∆*R*² = .05, *p* < .001)  Age  Gender  Education Dummy (high school)  Education Dummy (university degree)  Main Evening News (ORF)  Main Evening News (Servus TV)  Newspapers (Print or Online)  Social Media Posts (Friends & Family)  Social Media Posts (Celebrities)  Social Media Posts (News Media)  ORF News-Commentary Dummy (only once)  ORF News-Commentary Dummy (sometimes)  ORF News-Commentary Dummy (always)  Servus TV News-Commentary Dummy (only once)  Servus TV News-Commentary Dummy (sometimes)  Servus TV News-Commentary Dummy (always) |  | 0.01  –0.15  0.30  0.24  0.01  –0.14  0.17  0.09  0.09  –0.08  0.04  –0.02  0.01  0.01  0.01  –0.08  0.18  0.06  0.03  –0.01  0.04  –0.02  0.01  0.02  0.13  0.48  0.90  –0.05  –0.54  –1.45 | <0.01  0.10  0.11  0.13  <0.01  0.09  0.11  0.12  0.01  0.01  0.01  0.01  0.02  0.01  <0.01  0.09  0.10  0.12  0.01  0.02  0.01  0.01  0.02  0.01  0.16  0.13  0.19  0.15  0.13  0.21 | .13  –.05  .09  .06  .05  –.04  .05  .02  .30  –.18  .15  –.08  .02  .05  .05  –.03  .05  .01  .11  –.03  .14  –.07  .02  .05  .03  .15  .24  –.01  –.13  –.22 | <.001  .129  .008  .064  .162  .128  .112  .459  <.001  <.001  <.001  .022  .623  .225  .150  .349  .077  .623  .014  .367  <.001  .034  .486  .192  .415  <.001  <.001  .743  <.001  <.001 |
| *Note.* Gender: 1 = female, 2 = male | | | | | |

**Table OSM 6**

| *Hierarchical Multiple Regression Analyses Predicting Compliance with Government Measures from “Alarmist” (ORF) and “Reassurance” (Servus TV) News-Commentary Exposure (Study 2)* | | | | | |
| --- | --- | --- | --- | --- | --- |
| *B* SE β *p* | | | | | |
| *Step 1: Demographics (*∆*R*² = .04, *p* < .001)  Age  Gender  Education Dummy (high school)  Education Dummy (university degree)  *Step 2: Media Variables (*∆*R*² = .06, *p* < .001)  Age  Gender  Education Dummy (high school)  Education Dummy (university degree)  Main Evening News (ORF)  Main Evening News (Servus TV)  Newspapers (Print or Online)  Social Media Posts (Friends & Family)  Social Media Posts (Celebrities)  Social Media Posts (News Media)  *Step 3: News Commentary* (∆*R*² = .02, *p* < .001)  Age  Gender  Education Dummy (high school)  Education Dummy (university degree)  Main Evening News (ORF)  Main Evening News (Servus TV)  Newspapers (Print or Online)  Social Media Posts (Friends & Family)  Social Media Posts (Celebrities)  Social Media Posts (News Media)  ORF News-Commentary Dummy (only once)  ORF News-Commentary Dummy (sometimes)  ORF News-Commentary Dummy (always)  Servus TV News-Commentary Dummy (only once)  Servus TV News-Commentary Dummy (sometimes)  Servus TV News-Commentary Dummy (always) |  | 0.01  –0.28  0.03  0.07  0.01  –0.30  –0.03  <0.01  0.05  –0.03  0.02  –0.01  –0.02  0.01  0.01  –0.27  –0.03  –0.02  0.02  –0.00  0.02  –0.01  –0.02  0.02  0.18  0.31  0.45  –0.11  –0.17  –0.69 | <0.01  0.07  0.08  0.09  <0.01  0.07  0.08  0.09  0.01  0.01  0.01  0.01  0.01  0.01  <0.01  0.07  0.08  0.09  0.01  0.01  0.01  0.01  0.01  0.01  0.12  0.10  0.14  0.11  0.10  0.16 | .16  –.12  .01  .03  .08  –.13  –.01  <.01  .20  –.09  .11  –.04  –.07  .06  .09  –.12  –.01  –.01  .08  –.01  .10  –.03  –.07  .06  .05  .13  .16  –.03  –.06  –.14 | <.001  <.001  .771  .436  .015  <.001  .706  .994  <.001  .003  <.001  .300  .060  .116  .014  <.001  .717  .826  .100  .788  .001  .338  .071  .098  .158  .002  .002  .319  .099  <.001 |
| *Note.* Gender: 1 = female, 2 = male | | | | | |

**Figure OSM 1**

*Difference in Pre-Measured Perceptions, Political Attitudes, and Compliance Intentions Between Forced Viewers and Self-Selectors Before Starting to Process the News Item*


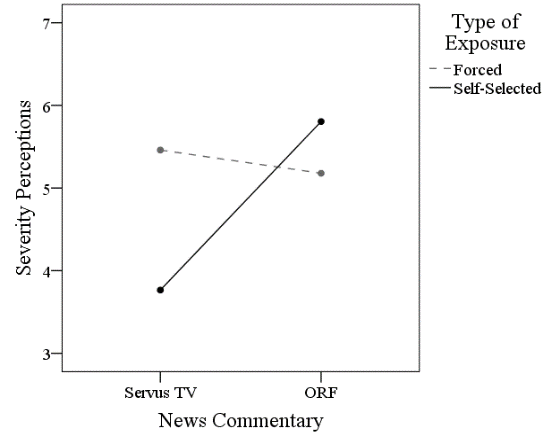

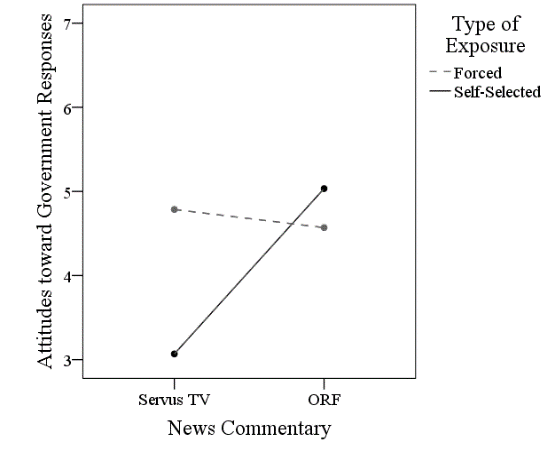

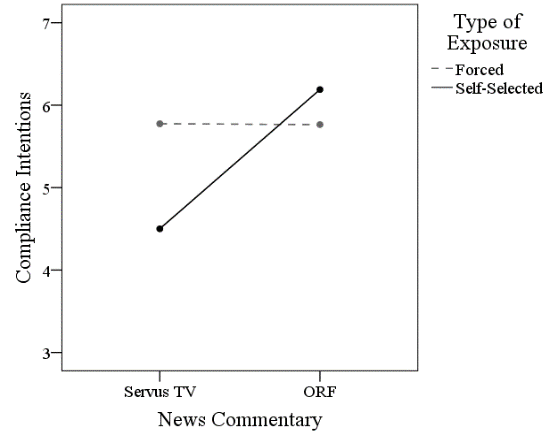


**Table OSM 7**

*Transcripts of the Video Material Used in Study 3*

**ORF Video**

Pediatrics in Austria are tirelessly emphasizing that kids are not more infectious than adults. This is indeed the case. But not less infectious, one should add. That means that it is indeed possible that infections are carried in like this. And if we, now, cannot trace more than three quarters of the cases, then we’re at the point where one has to say we can’t exclude anything at all. And hence, schools cannot be an exception either. And we have to say we have to substantially reduce the overall number of contacts. And if 1.3 million pupils are taken out of the public sphere, and, subsequentially, the parents who take the children to school, this obviously causes a crucial reduction in contacts. We saw this in the spring; this has an enormous impact relatively quickly. That’s why this measure is quite reasonable.

We see, obviously, in movement profiles, that this has been unsuccessful so far, that people don’t comply too well. When we speak of a reduction of 30%, then that’s not sufficient; hence, the number of people, the number of contacts, that can be deduced from the movement profiles, where one can easily see via the mobile phone networks how much people move. This number is still clearly too high. Hence, a lockdown can be quite beneficial. And this should be reflected in the numbers.

I believe it is also a bit necessary to use our own brains. Seeing the run on shopping malls today—and business revenues are duly granted that, by all means—but today, there is only a bulk discount for the virus. It is barely comprehensible why thousands of people want to bustle around here, while we know we have a level of endemic infection that lets us assume one or another superspreader will be among us. What can be scored here is an infection, at most. However, this is exactly the kind of personal responsibility that we counted on in the last weeks and that didn’t work too well.

One can try to imagine the situation of having three patients, but only having one bed left for one of these patients, having to decide which two people will not be connected to the necessary medical devices, because we simply don’t have any availability anymore. This is the infamous “triage.” Something resembling a catalog has been introduced in the spring: how to proceed based on which criteria, but we know in these critical situations—I have personally spoken with intensive care physicians—most of the time these decisions are based on age. Older people are not at the top of this list. However, they try to treat those, of course, for whom treatment still makes the most sense. But those are really, really hard decisions that no one actually wants physicians to make.

The additional problem is that a lot of these COVID-patients have to spend a very long time in intensive care units: 14 days, and some remain there for 50/60 days in these intensive care units. They have to be turned over onto their stomachs, for example—just a quick example—it takes five nurses to accomplish this and these are highly qualified staff. Klaus Markstaller compared it to a pilot in an airplane today: One can’t just ask anyone to do this, because these intensive care units utilize highly complicated medical devices and naturally highly skilled staff are needed to meet these very difficult challenges.

And something that is very important: We now have eleven vaccines in phase three—already pointing toward approval—which all follow the same train of thought: They all target the same surface protein of the virus, which is technically the dangerous part that helps the virus enter the cells. However, this is also the weak spot of this virus. And all these vaccines aim exactly at this and that’s our hope.

**Servus TV Video**

Even though there are still no reliable data about people who tested positive, on actual sick people, on the main contagion routes, and available beds in the so-called second wave, mathematicians and other theoreticians provide daily prognoses in this dense fog. Factually, prognoses are based on missing numbers, and due to this reputable fog-prognosis, they impose lockdowns and multiple other reasonable measures that shut down public social life and the economy and partially ruin them permanently. And all this is obviously “for the good of all.”

Everybody has to make sacrifices as ordered by the government and—now—has to pay for the government’s systematical skimping on the healthcare system over the years. Recent press reports show how dramatic the situation is in Austrian and German clinics: “Vienna’s hospitals are this crowded,” which reports on precarious situations in Viennese hospitals, where heaps of patients lie in intensive care units and in corridors because the rooms are thoroughly overcrowded. The number of corridor beds is not revealed. The President of the Medical Chamber, Thomas Szekeres, speaks of mismanagement and the Head of the Viennese ÖVP Gernot Blümel criticizes: “In Vienna, corridor beds are the rule all year long, rather than the exception during flu season.” Yes, ladies and gentlemen, I am being told right now [touches his ear, simulates being spoken to via an in-ear headphone] that this article from the *Krone* is from 2017.

“Viennese hospitals struggle with overload,” *Der Standard* writes in a headline and reports that the occupancy rate in Vienna exploded within just two weeks. *Die Presse* also reports on shortages in Viennese hospitals and patients who have to be treated in corridors. But you can already guess that these worrisome articles were not drafted in 2020, but in 2012. Strange though, that the annual flu obviously creates absolute emergency situations in our hospitals every two to three years, without reactions from politicians and without forcing lockdowns and other repressive measures on the country as a doubtful benefit.

Well, this year we still have below-average mortality rates in Austria and Germany. That means, on average, less people died in 2020 than usual—despite the coronavirus—not to mention the bad “flu winters,” for example 2017/2018, when influenza consumed 25,000 people in Germany alone. Respectively, it is understandable that the government now counts on panic, panic, panic, and that the quality media plays perfectly along with the fearmongering with new horrific numbers daily and pictures from intensive care units.

The fact that the Swedish economy looks a lot better compared to ours because they have no lockdowns and few restrictions, but a lot of personal responsibility for its citizens—90% of whom stand behind their government’s COVID-policies—is not reported, as is the case for the fact that Sweden has been much better at coping with the COVID crisis since June than most other countries and is mourning substantially fewer COVID deaths in the last few weeks than Austria, for example.

All right, but we have a government that manages to blindly steer through the COVID fog, closing restaurants, outdoor dining, coffee shops, zoos, businesses, schools, and many more.

Many people are pleased to notice that the increasing infringements on basic civil rights still do not receive attention in the quality media and worried critics are accused of coronavirus denial without any differentiation, while the leftist journalist clan has been worrying about a possible infringement on the rights of Islamic terrorists and murderers for days. Not without spreading repetitive, uncritical reports of jubilation about the salvaging vaccination from time to time, which is supposed to facilitate a life in the much-vaunted “new normal” after corona.

**Control Video** (Source: *Die Techniker Krankenkasse*, Dr. Johannes Wimmer, https://www.youtube.com/watch?v=bmqFN8cFjzg)

How much water is one actually supposed to drink a day? What are a doctor’s best tips for drinking enough at the office or at work? It’s not that easy to answer. The most important thing is, however, no doubt about it—before we start talking about any numbers: Water is essential for the body. The body consists of more than 60% water. We can’t even imagine that more than half of the body sitting in front of you is water, even if it sounds like it sometimes. And the next thing is: Every process in the body requires water. No matter if you want to wash out toxins, the performance of organs, blood pressure, even the cells need water. Everything works solely with water. That’s why the same amount of water we lose has to enter the body as well. We don’t just lose water on the toilet when we pee, but we also lose water with every breath we take, and we transpire a lot of water at night, that’s when it starts. About half a liter/a liter and a half is what we transpire at night. During the day, depending on how hard I work and what I do—sports etc. —I also lose water. And this wants to be replaced.

That’s when we say, on average, drinking three liters of water a day, that should be good, if you are not a professional athlete or something like that. Athletes know this and they feel it too. When they say I am on the move with the racing bike, or I do whatever, or I lift weights, or whatnot—and this also depends on the diet—then I need more water. Thus, when you listen to your body, this works well.

The body also signals the good old feeling of thirst. This is not a warning signal. Not to say, “Oh my God, now I’m thirsty and soon everything will be over,” no, this is completely normal. Whenever I am thirsty, I should drink something. We forget this often, because we sit in front of the computer, or we have something really important and tap-tap-tap, we didn’t drink anything all day. And you want to get up and then realize, somehow, I feel dizzy. This is, for example, a typical problem that occurs when you drink too little because the body doesn’t have enough water to retain the blood pressure. Therefore, put a bottle of water or a jug on your desk, drink something, and not just coffee, coke, and random sodas, okay? That is the problem. Coffee, as yummy as it is, contains caffeine. And I, for example, react very strongly to caffeine and pee a lot. That is different in all people—there are people who can drink coffee by the pitcher and others are affected right away. Therefore, in Italy, there is a glass of water with the espresso to make up for this. Important: The problem in many offices is that you get some coffee in the morning and you stick with it throughout the day. A coke for lunch too, that’s bad. So, take note. drink water. Alcohol is exactly the same: In the evening, when I go out and have a beer with coworkers—the best thing would be to have a cup of water with every beer or wine. Then the body is lubricated like a machine and runs the way it is supposed to run without—so to speak—biting off more than it can chew.

Apart from that, also keep in mind not to drink too much. Not making yourself drink it, “Oi, I have to somehow drink this,” because this can also be dangerous, the body may not cope well with this. So, just find a normal amount that is something around three liters. With fruits and vegetables, you also ingest a lot of water. Meaning that, if I eat a good soup, I might not need to drink as much water as when I have a big Schnitzel, fries, and salad, which is not that healthy anyways.

So, always bring water. The harder you work, the more you need. Also keep this in mind when exercising and then everything should slip, slide, and roll as smoothly as it is supposed to.

**Table OSM 8**

*Additional Analyses (Study 3): Controlling for Prior News-Commentary Use Did Not Alter the Results in a Substantial Way*

Based on the suggestion by an anonymous reviewer, we re-ran the regression models reported in study 3’s “selective exposure” and “preference-based reinforcement” sections. We tested whether the predictive power of perceptions, attitudes, and compliance intentions decrease when prior news-commentary use is statistically controlled for when predicting news-commentary choice. Importantly, controlling for prior news-commentary use (i.e., ORF and Servus TV—we used the same measure as in study 2; please see study 2’s method section) did not change the results in a substantial way:

The results for the section on selective exposure (i.e., separate hierarchical binary logistic regression models, controlling for age, gender, education, and political orientation) *without* prior news-commentary use (as reported in the manuscript):

- Perceived threat severity, *B* = 1.00, *SE* = 0.13, *Wald* = 61.21, *df* = 1, *Odds ratio* = 2.73, *p* < .001
- Attitudes toward government responses, *B* = 0.94, *SE* = 0.12, *Wald* = 61.78, *df* = 1, *Odds ratio* = 2.57, *p* < .001
- Compliance, *B* = 0.81, *SE* = 0.12, *Wald* = 46.88, *df* = 1, *Odds ratio* = 2.25, *p* < .001

The results for the section on selective exposure (i.e., separate hierarchical binary logistic regression models, controlling for age, gender, education, political orientation, and prior news-commentary use) *with* prior news-commentary use (additional analysis based on R1’s comment): (nearly identical coefficients)

- Perceived threat severity, *B* = 0.95, *SE* = 0.15, *Wald* = 11.22, *df* = 1, *Odds ratio* = 2.59, *p* < .001
- Attitudes toward government responses, *B* = 0.90, *SE* = 0.14, *Wald* = 43.22, *df* = 1, *Odds ratio* = 2.46, *p* < .001
- Compliance, *B* = 0.67, *SE* = 0.12, *Wald* = 28.89, *df* = 1, *Odds ratio* = 1.95, *p* < .001

The results for the section on preference-based reinforcement (i.e., multiple regression models, controlling for age, gender, education, political orientation, and prior perceptions/attitudes/compliance; conceptual variable = news choice) *without* prior news-commentary use (as reported in the manuscript):

- News choice was a significant predictor for Perceived threat severity measured after exposure, *B* = 0.60, *SE* = .10, β = .15, *t* = 6.32, *p* < .001
- News choice was a significant predictor for Attitudes toward government responses measured after exposure, *B* = 0.49, *SE* = .08, β = .13, *t* = 6.38, *p* < .001
- News choice was a significant predictor for Compliance measured after exposure, *B* = 0.21, *SE* = .07, β = .06, *t* = 3.10, *p* = .002

The results for the section on preference-based reinforcement (i.e., multiple regression models, controlling for age, gender, education, political orientation, prior news-commentary use, and prior perceptions/attitudes/compliance; conceptual variable = news choice) *with* prior news-commentary use (additional analysis based on R1’s comment): (nearly identical coefficients)

- News choice was a significant predictor for Perceived threat severity measured after exposure, *B* = 0.55, *SE* = .10, β = .14, *t* = 5.57, *p* < .001
- News choice was a significant predictor for Attitudes toward government responses measured after exposure, *B* = 0.46, *SE* = .08, β = .12, *t* = 5.64, *p* < .001
- News choice was a significant predictor for Compliance measured after exposure, *B* = 0.20, *SE* = .07, β = .06, *t* = 2.72, *p* = .007

Additional analyses showed that perceptions, attitudes, and compliance predicted news choice. Prior news-commentary use did not substantially alter the results.
